# Portable comprehensive two-dimensional micro-gas chromatography using an integrated flow-restricted pneumatic modulator

**DOI:** 10.1038/s41378-022-00452-5

**Published:** 2022-11-01

**Authors:** Xiaheng Huang, Maxwell Wei-hao Li, Wenzhe Zang, Xiaolu Huang, Anjali Devi Sivakumar, Ruchi Sharma, Xudong Fan

**Affiliations:** 1 Department of Biomedical Engineering, University of Michigan, Ann Arbor, MI 48109 USA; 2 Department of Electrical Engineering and Computer Science, University of Michigan, Ann Arbor, MI 48109 USA; 3 Center for Wireless Integrated MicroSensing and Systems (WIMS2), University of Michigan, Ann Arbor, MI 48109 USA; 4 Max Harry Weil Institute for Critical Care Research and InnovationUniversity of Michigan, Ann Arbor, MI 48109 USA

**Keywords:** Electrical and electronic engineering, Chemistry

## Abstract

Two-dimensional (2D) gas chromatography (GC) provides enhanced vapor separation capabilities in contrast to conventional one-dimensional GC and is useful for the analysis of highly complex chemical samples. We developed a microfabricated flow-restricted pneumatic modulator (FRPM) for portable comprehensive 2D micro-GC (μGC), which enables rapid ^2^D injection and separation without compromising the ^1^D separation speed and eluent peak profiles. ^2^D injection characteristics such as injection peak width and peak height were fully characterized by using flow-through micro-photoionization detectors (μPIDs) at the FRPM inlet and outlet. A ^2^D injection peak width of ~25 ms could be achieved with a ^2^D/^1^D flow rate ratio over 10. The FRPM was further integrated with a 0.5-m long ^2^D μcolumn on the same chip, and its performance was characterized. Finally, we developed an automated portable comprehensive 2D μGC consisting of a 10 m OV-1 ^1^D μcolumn, an integrated FRPM with a built-in 0.5 m polyethylene glycol ^2^D μcolumn, and two μPIDs. Rapid separation of 40 volatile organic compounds in ~5 min was demonstrated. A hybrid 2D contour plot was constructed by using both ^1^D and ^2^D chromatograms obtained with the two μPIDs at the end of the ^1^D and ^2^D μcolumns, which was enabled by the presence of the flow resistor in the FRPM.

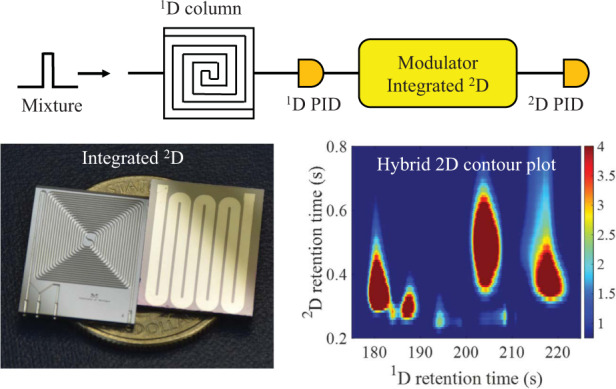

## Introduction

Microfabricated gas chromatography (μGC) is a powerful portable vapor analysis method for applications such as environmental protection and monitoring, workplace hazard analysis, and biomedicine^1–5^. To date, nearly all μGC devices are one-dimensional (1D) GC with relatively short columns (<10 m), which limits the separation performance for complex mixtures for many field applications^6–9^ (e.g., petroleum, food, metabolomic or forensic), which may require the analysis of hundreds of diverse compounds. Thus, the addition of a second column in two-dimensional (2D) GC, such as heart-cutting or comprehensive 2D GC, is needed to further enhance the separation capabilities and broaden the range of compounds that can be analyzed by a single portable μGC device.

In comprehensive 2D GC, a modulator is critical for periodically cutting portions of eluents from the first-dimensional (^1^D) column and injecting them into the second-dimensional (^2^D) column for further analysis^10,11^. These modulators are typically either thermal or pneumatic. A thermal modulator first traps a portion of an eluent from the ^1^D column, injects the trapped eluent into the ^2^D column by rapidly raising the temperature, and is then cooled immediately to trap subsequent eluents from the ^1^D column. The major drawbacks of the thermal modulator are the need for (1) high power for rapid temperature ramping and (2) rapid cooling mechanisms (such as liquid N_2_ or liquid CO_2_). These factors increase the modulator footprint and therefore are not conducive to μGC development. While a microfabricated thermal modulator using thermal-electric cooling was recently demonstrated^12,13^, its operation was still power intensive, it was difficult to fabricate and maintain, and it was incapable of trapping light compounds.

A pneumatic modulator uses external valves and auxiliary flows to inject a portion of an eluent from the ^1^D column into the ^2^D column without rapid heating or cooling. Commonly used types of modulators include the stop-flow modulator^14–16^, in which the flow in ^1^D is suspended temporarily when ^2^D separation takes place. While the stop-flow modulator (essentially a T-junction) can be microfabricated^15^, the use of the stop-flow mode significantly increases the ^1^D separation time^11,16^ and causes additional peak broadening. Another commonly used pneumatic flow switching modulator is the Deans switch^11,17–20^, which has been microfabricated for comprehensive 2D GC^18^. While the Deans switch allows for continuous ^1^D separation concomitant with ^2^D separation, the flow rates in ^1^D and ^2^D need to be carefully adjusted to avoid backflow in ^1^D. A high flow rate ratio between ^2^D and ^1^D results in disturbances in ^1^D flow. Consequently, it is difficult to achieve sharp ^2^D injection and rapid ^2^D separation. In addition, the analyte concentration in ^2^D is diluted due to the auxiliary flow needed to transfer the ^1^D eluent to ^2^D. Differential flow modulators^10,11,21,22^ use 4- or 6-port valves so that the ^1^D and ^2^D flows are independent and thus allow for concomitant ^1^D and ^2^D separation while permitting a high ^2^D to ^1^D flow rate ratio for sharp ^2^D injection and improved ^2^D separation. However, 4- and 6-port valves are very bulky and heavy, which are unsuitable for μGC.

This work demonstrates a microfabricated chip-based flow-restricted pneumatic modulator (FRPM) enabling sharp ^2^D injection and a high ^2^D flow rate without suspending ^1^D separation. The design, fabrication, and characterization of this pneumatic modulator are described, and an injection peak width of ~25 ms is achieved at a ^2^D/^1^D flow rate ratio over 10 without ^1^D perturbation. Subsequently, the FRPM is monolithically integrated with a 0.5 m ^2^D column on a single chip. Finally, a first-of-its-kind portable automated comprehensive 2D μGC device is developed, consisting of a 10 m OV-1 ^1^D microfabricated column (μcolumn), an integrated FRPM with a built-in 0.5 m WAX (i.e., polyethylene glycol (PEG)) ^2^D μcolumn, and two flow-through micro-photoionization detectors (μPIDs). The entire device was run standalone without any benchtop components. The rapid separation of 40 volatile organic compounds (VOCs) in 5 min is demonstrated. A 2D contour plot is constructed by using both ^1^D and ^2^D chromatograms obtained with the two μPIDs at the end of the ^1^D and ^2^D μcolumns, showing improved peak capacity compared to that of conventional comprehensive 2D GC using only one vapor detector at the end of the ^2^D column. The use of the μPID at the end of ^1^D is crucially enabled by the presence of the flow resistor in the FRPM.

## FRPM design and principles

**Fig. 1:**
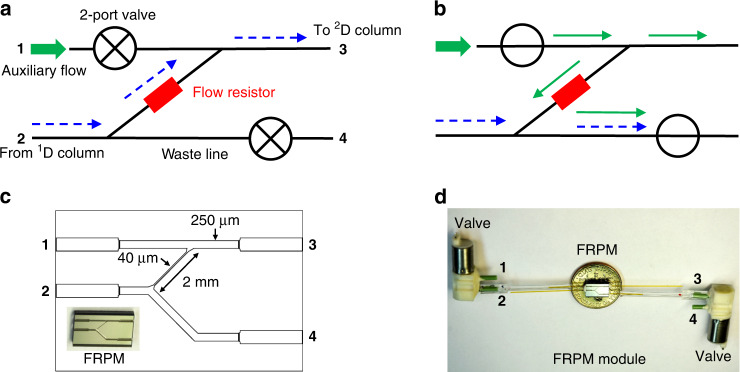
Flow-restricted pneumatic modulator (FRPM) operation for comprehensive 2D μGC. **a** ^1^D to ^2^D loading configuration with both valves closed (typical flow rate: ~1 mL/min). The blue arrows depict the ^1^D flow direction. **b** ^2^D separation with both valves open for high ^2^D flow (typical flow rate: ~10 mL/min), enabling sharp ^2^D injection and rapid ^2^D separation. The green arrows depict the auxiliary flow direction. **c** Microfabricated FRPM schematic and photograph. The flow resistor is a narrow channel with a cross-section of 40 μm × 170 μm (width × depth) and a length of 2 mm. All other channels have cross-sections of 250 μm × 250 μm (width × depth). **d** Photograph of the FRPM module with the FRPM and two 2-port valves. Ports 1–4 are labeled on the schematics and photographs

A block diagram for the FRPM along with its operation is provided in Fig. 1. The FRPM consists of an inlet for auxiliary flow (Port 1), an inlet for ^1^D eluents (Port 2), an outlet connected to the ^2^D column (Port 3), and an outlet as the waste line (Port 4), as well as an internal flow resistor between ^1^D and ^2^D. The auxiliary flow and waste line are controlled by two 2-port valves. During loading (Fig. 1a), both valves are closed, and a portion of the ^1^D eluent is loaded onto the ^2^D column through the flow resistor. During ^2^D separation (Fig. 1b), both valves are open, and a high auxiliary flow simultaneously provides the ^2^D carrier gas flow for ^2^D separation and the buffer flow that prevents the ^1^D eluent from entering the ^2^D column. Concurrently, ^1^D separation continues, and the ^1^D eluent is diverted to the waste line. After ^2^D separation, both valves are closed again, and a new modulation cycle begins. The fabrication of the FRPM is shown in Supplementary Figs. S1 and S2.

**Fig. 2:**
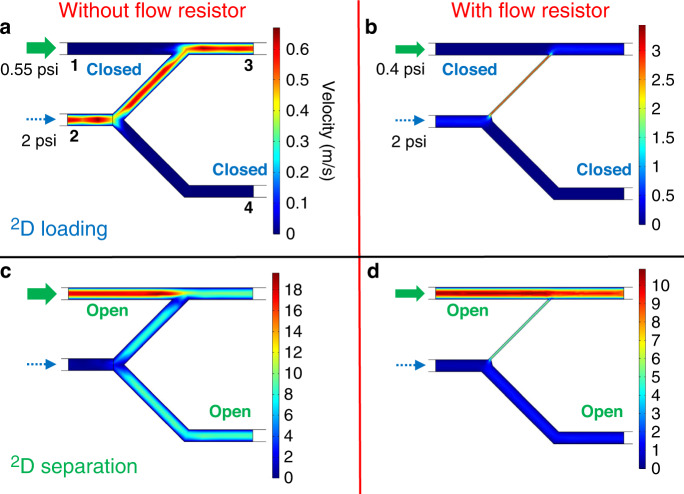
Microfluidic simulations of the FRPM module. Flow velocity field distribution of the FRPM module during ^2^D loading and separation phases without (**a**, **c**) and with (**b**, **d**) a flow resistor. The simulated geometry of the FRPM module is the same as that shown in Fig. 1c. The entire simulation geometry includes a 10 m column with a channel width of 250 μm (not shown) attached to the FRPM module at Port 2. Input pressures are assigned at the inlet of the 10 m column (2 psi) and at the inlet of Port 1 in the FRPM module (0.55 psi for the FPRM without a flow resistor (**a**, **c**) and 0.4 psi for the FRPM with a flow resistor (**b**, **d**)

Computational fluid dynamics simulation was performed to examine the theoretical flow inside the FRPM. During ^2^D loading (Fig. 2a, b), the ^1^D to ^2^D flow velocity (at Port 3) is the same regardless of the presence of the flow resistor because the additional flow resistance resulting from the 40-μm narrow channel of the FRPM is negligible compared to that of the upstream 10 m column. During ^2^D separation, the FRPM without a flow resistor requires a higher input pressure from Port 1 (0.55 vs. 0.4 psi) to maintain the same flow rate at Port 3 as that of the FRPM with a flow resistor (see Fig. 2c, d). A comparison between Fig. 2c and d shows that during ^2^D separation, the flow resistor causes more of the auxiliary flow from Port 1 to be diverted to ^2^D (as the ^2^D carrier gas), whereas much of the auxiliary flow is flushed downward away from ^2^D when the flow resistor is not present. In both cases, the ^1^D flow is diverted to the waste line by the buffer flow passing through the ^1^D to ^2^D connection channel, which prevents the ^1^D flow from entering ^2^D. With the flow resistor, ^1^D separation continues without significant interruption, but without the flow resistor, significant flow perturbations are observed when the high flow from the auxiliary line causes flow shocks in ^1^D.

Compared to the other aforementioned pneumatic modulators, the FRPM modulator has several advantages.

(1) A high auxiliary flow rate can be used for sharp ^2^D injection and rapid ^2^D separation.

(2) The flow resistor restricts the auxiliary flow that is spent on the waste line (see Fig. 2), which saves the auxiliary flow.

(3) Again, due to the flow resistor, the impact of the auxiliary flow on the ^1^D flow and separation is minimized. Consequently, a large range of auxiliary flow rates and ^1^D flow rates can be selected without ^1^D flow perturbation (such as flow shocks upon modulation switching and ^1^D backflow). This allows for detection immediately after the ^1^D outlet to directly monitor ^1^D separation, which enables utilization of the ^1^D chromatogram for constructing 2D contour plots (as further discussed later).

(4) The eluent concentration (or density) at the transfer junction from the ^1^D outlet to the ^2^D inlet is preserved. In contrast, the Deans switch relies on the auxiliary flow to push the eluent from ^1^D to ^2^D, consequently diluting the eluent concentration and reducing the ^2^D signal when a concentration-dependent vapor detector (e.g., PID) is used.

(5) ^1^D separation is continuous (unlike in stop-flow modulation), which expedites ^1^D separation and reduces ^1^D peak broadening.

(6) The FRPM is versatile and can be operated in stop-flow mode by permanently closing the waste line valve and letting the ^1^D and auxiliary flow share the same pressure/flow source (see Discussion).

(7) The FRPM can be easily microfabricated and even integrated with a ^2^D column on a single chip.

## Results

### FRPM module

FRPM chips were microfabricated using the same process as that for μcolumns (details in Supplementary Fig. S1). Each FRPM chip has dimensions of 8 mm × 5 mm × 1 mm (length × width × thickness). Figure 1c illustrates a schematic of the microfluidic channels inside the FRPM, with a 2-mm long, 40 μm × 170 μm (width × depth) channel as the built-in flow resistor. The flow resistor’s width and depth can be adjusted to achieve different flow resistances. All other channels have cross-sections of 250 μm × 250 μm. FRPM modules were constructed by connecting the FRPM chip to two 2-port valves at the corresponding ports (Fig. 1d).

**Fig. 3:**
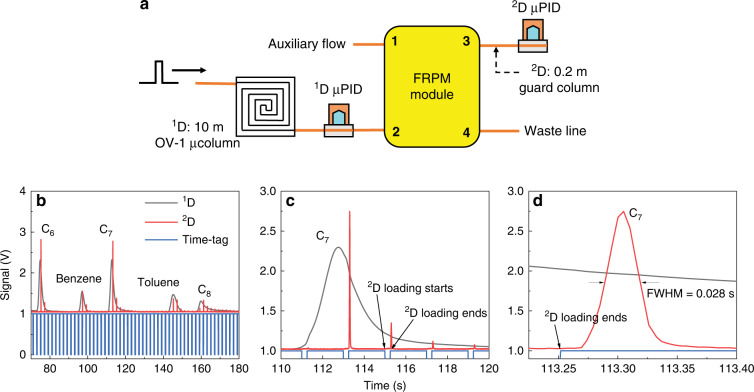
Operation characteristics of the FRPM module. **a** Setup used to characterize the FRPM module. The sample mixture is injected into a 10 m OV-1 coated microcolumn (μcolumn) with a cross-section of 200 μm × 250 μm (width × depth). A microphotoionization detector (^1^D μPID) is connected to the ^1^D μcolumn and used to monitor the ^1^D eluents. The ^2^D μPID is connected to the outlet of the FRPM module via a 20 cm guard column (inner diameter: 250 μm) and used to monitor the ^2^D eluents. Ports 1–4 labeled on the FRPM module are described in Fig. 1(A). Unmodulated operation is shown in (**a**). **b** ^1^D and ^2^D chromatograms of C_6_, C_7_, C_8_, benzene, and toluene. The time-tags (shown as a blue square wave) record the closed (= 0) and open (= 1) states of the FRPM module valves, corresponding to ^2^D loading (= 0) and ^2^D separation (= 1), respectively. **c** Magnification of C_7_ peak in ^1^D and ^2^D. **d** Magnification of C_7_ peak in ^2^D. Experimental conditions: loading time = 0.25 s; modulation time = 2 s; ^1^D flow rate = 1.2 mL/min (measured at Port 3 with both valves closed). The flow rate is nearly the same when measured at the outlet of ^1^D (before the FRPM), indicating that the impact of the flow resistor on the ^1^D flow is negligible. ^2^D flow rate = 20 mL/min (measured at Port 3 with both valves open). The ^1^D μcolumn temperature ramping profile is provided in Fig. S3(B). The FRPM is kept at the isothermal ambient temperature (~20 °C). Helium is used as both the ^1^D carrier gas and auxiliary flow

As illustrated in Fig. 3a, ^2^D injection using the FRPM module was characterized by only a 10 m OV-1 ^1^D μcolumn and a 20 cm guard column in ^2^D (no ^2^D separation column). Two flow-through μPIDs were used to measure and compare eluents immediately before and after the FRPM. Initial characterization was carried out using unmodulated operation (chromatograms in Supplementary Fig. S3a). All ^1^D eluents were transferred to ^2^D with slight delays between the eluent peaks detected by the ^1^D and ^2^D μPID, which increased for heavier compounds. These delays resulted from the 20 cm guard column in ^2^D. A comparison of the C_6_ and C_7_ peak heights showed that the ^2^D μPID is approximately 2.4 times more sensitive than the ^1^D μPID. The relative peak heights for other compounds in the ^2^D μPID are reduced compared to those in ^1^D again due to peak broadening resulting from the 20 cm guard column.

**Fig. 4:**
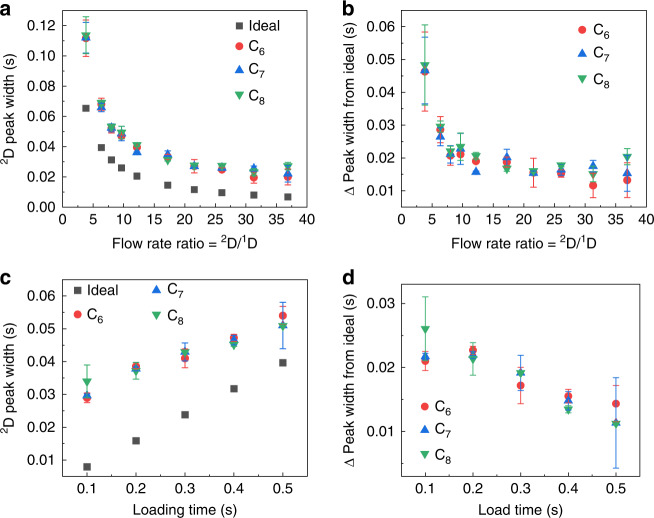
^2^D peak width dependence on ^2^D/^1^D flow rate ratio and loading time. ^2^D peak widths (full width at half maximum) of C_6_, C_7_, and C_8_ detected by ^2^D μPID vs. ^2^D/^1^D flow rate ratio (**a**) and loading time (**c**). The ideal ^2^D peak width is calculated by the loading time divided by the flow rate ratio. Deviations of ^2^D peak widths from ideal injection widths vs. flow rate ratio (**b**) and loading time (**d**). In (**a**) and (**b**), the loading time is fixed at 0.25 s and the ^2^D flow rate varies from 4 to 40 mL/min. In (**c**) and (**d**), the ^2^D flow rate is fixed at 16 mL/min and the loading time varies from 0.1 to 0.5 s. In all experiments, ^1^D flow rate = 1.2 mL/min and modulation time = 2 s. Error bars are obtained with 3 measurements

Modulated operation was investigated next. Figure 3b–d shows an example of ^1^D and modulated ^2^D chromatograms using alkanes and aromatics. The heights of the modulated ^2^D peaks are lower than those for the unmodulated peaks because the loading from ^1^D to ^2^D may not occur exactly at the apexes of the ^1^D peaks. Since sharp ^2^D injections are crucial for maximizing the ^2^D peak capacity, the ^2^D injection peak width (defined as the full-width-at-half-maximum) for C_6_, C_7_, and C_8_ as a function of the flow rate ratio between ^2^D and ^1^D (^2^D/^1^D) was examined (Fig. 4a). In general, the injection peak width decreases with an increasing ^2^D/^1^D flow rate ratio. However, the measured injection peak width is always broader than the ideal peak width (defined as the loading time divided by the ^2^D/^1^D flow rate ratio). This broadening is caused by the 20 cm guard column, and the broadening vs. flow rate can be viewed as the Golay plot of said column (Fig. 4b). The ^2^D injection peak width is also affected by loading time and is characterized in Fig. 4c at a fixed flow rate ratio of 13. The measured peak width increases linearly with increasing loading time and is again broader than the theoretical value. The broadening effect diminishes with longer loading times (Fig. 4d) since the broadening from the guard column becomes less prominent. Section S2 (Supplementary Figs. S4–S11) of the Supplementary Information provides the maximally allowed ^2^D/^1^D flow ratio without affecting the ^1^D flow and peak height (and peak area) for different loading times. In addition, we experimentally tested a series of flow resistors with widths of 20, 60, 80, and 250 μm and corresponding depths of 160, 190, 210, and 250 μm (see Supplementary Fig. S6 for the results of an FRPM with a 250-μm width). The different depths occur due to reactive ion etching lag during the deep reactive ion etching process. Considering an appropriately high flow rate ratio of ~10, ^1^D flow jittering is eliminated only once the flow resistor’s width is reduced to 40 μm (depth = 170 μm). A width of 20 μm also eliminates the ^1^D jittering but is prone to channel blocking during microfabrication. Based on these results, we selected the FRPM chip with a flow resistor width × depth = 40 μm × 170 μm, which allowed for an injection peak as sharp as ~25 ms achieved with a loading time of 0.25 s and a flow rate ratio larger than 10. This was accomplished without perturbing the ^1^D flow or significantly slowing down ^1^D separation due to the sufficiently high flow resistance of the 40-μm channel.

An alternative FRPM module design replaces the two 2-port valves with a single 3-port valve (Supplementary Fig. S12). During ^2^D loading and separation, the 3-port valve directs the auxiliary flow to its normally opened and closed ports, respectively, allowing performance like that of the two-valve module (Supplementary Figs. S13 and S14). Compared to the two-valve configuration, the single-valve FRPM module uses fewer components and is thus cheaper and easier to maintain. However, the eluent concentration (or density) at the transfer junction from the ^1^D outlet to the ^2^D inlet is slightly reduced because of the additional buffer flow added to the ^1^D eluents during loading.

### Integrated FRPM and ^2^D μcolumn module

**Fig. 5:**
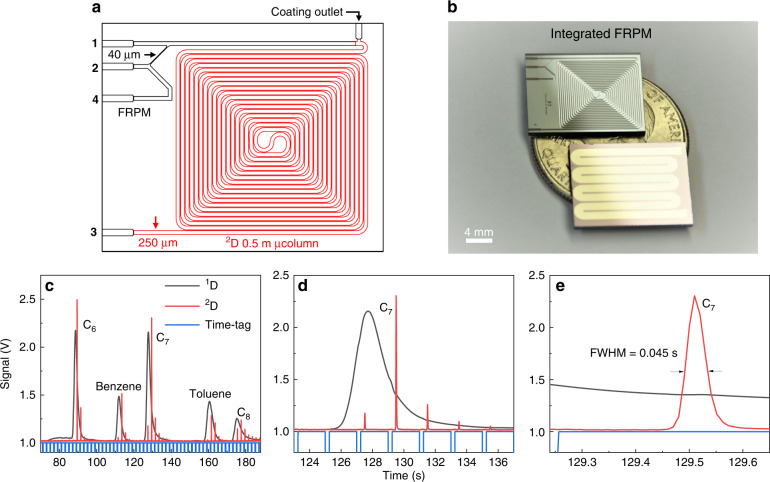
Operation characteristics of the ^2^D μcolumn integrated FRPM module. **a** Schematic of FRPM integrated with 0.5 m ^2^D μcolumn. **b** Photograph of integrated FRPM chip (top) with backside heater (bottom). **c** ^1^D and ^2^D chromatograms of C_6_, C_7_, C_8_, benzene, and toluene using deactivated integrated FRPM chip. **d** Magnification of C_7_ peak in ^1^D and ^2^D . **e** Magnification of C_7_ peak in ^2^D. Experimental conditions: loading time = 0.25 s; modulation time = 2 s; ^1^D flow rate = 1.1 mL/min; ^2^D flow rate = 14 mL/min. The ^1^D temperature ramping profile is the same as that in Figure 3. The integrated chip is at isothermal room temperature (~20 ^o^C). Helium is used as both ^1^D carrier gas and auxiliary flow

To further reduce the device footprint and number of interconnections, the FRPM was integrated with a 0.5 m ^2^D μcolumn (cross-section: 250 μm × 250 μm) on a single chip of dimensions 18 mm × 15 mm × 1 mm (length × width × thickness) (Fig. 5a, b). Because of the additional flow resistance from the 0.5 m ^2^D μcolumn, the integrated module was re-evaluated with the same methodology as the stand-alone module (Supplementary Figs. S15 and S16). As shown in Fig. 5c–e, at a flow rate ratio = 13, the integrated FRPM module demonstrates performance similar to that of the stand-alone module, with an additional ^2^D peak broadening of ~20 ms due to the extra 0.5 m μcolumn (the ^2^D μcolumn was only deactivated without any stationary phase coating yet).

### Automated portable comprehensive 2D μGC construction and operation

**Fig. 6:**
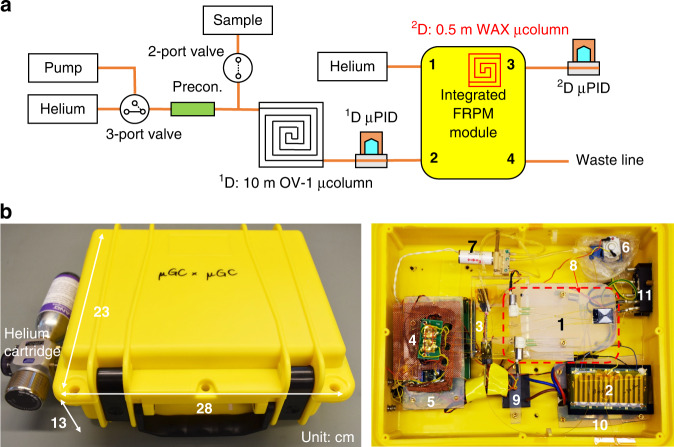
Portable comprehensive 2D μGC system. **a** Schematic of the integrated FRPM-based portable comprehensive 2D μGC device. The integrated FRPM module contains a 0.5 m long WAX ^2^D μcolumn. **b** System photographs. The device has dimensions 28 cm × 23 cm × 13 cm (length × width × height) and weighs 2.4 kg (including helium cartridge). (1) Integrated FRPM and 0.5 m WAX ^2^D μcolumn module (within the dashed square); (2) ^1^D 10 m OV-1 μcolumn; (3) Preconcentrator. (4) μPID array; (5) Printed circuit board and data acquisition card (copper mesh shielded); (6) Pump; (7) 3-port valve; (8) 2-port valve; (9) DC–DC converter; (10) 24 V power supply; (11) Rocker switch connected to wall power

We constructed a stand-alone automated portable comprehensive 2D μGC device (Fig. 6) consisting of a 10 m OV-1 ^1^D μcolumn (nonpolar), the integrated FRPM and 0.5 m WAX ^2^D μcolumn (polar), and two flow-through μPIDs at the ^1^D and ^2^D outlets, respectively, as well as accessories such as valves, a preconcentrator, a pump, helium cartridges, and in-house control software. Miniaturized comprehensive 2D GC at the subsystem level was investigated previously using μcolumns and thermal/pneumatic modulators^13,15^. However, these devices used benchtop GC injectors and/or detectors and were thus not automated stand-alone systems for field applications. This work presents a first-of-its-kind automated portable comprehensive 2D μGC without any benchtop components.

This comprehensive 2D μGC is different from traditional comprehensive 2D GC in a few aspects. First, traditional comprehensive ^2^D GC uses only one detector at the end of the ^2^D column. The ^1^D chromatogram is reconstructed based only on information from the ^2^D detector, which leads to errors in the ^1^D retention time, ^1^D peak broadening, and the possibility of undersampling of ^1^D peaks. In contrast, our comprehensive 2D μGC uses two flow-through μPIDs to monitor the ^1^D and ^2^D eluents. This arrangement removes the need for ^1^D chromatogram reconstruction, as the ^1^D chromatogram can be directly obtained from the ^1^D μPID. As a result, the ^1^D peak position (i.e., ^1^D retention time) is accurately determined, and the original ^1^D peak width is preserved, which improves the separation performance (i.e., peak capacity). Second, because of the two-detector arrangement, a new algorithm to generate hybrid 2D contour plots using both ^1^D and ^2^D chromatograms can be developed to improve the separation performance. Third, the modulation time is dynamically adjusted to accommodate different ^1^D peak widths. For example, a short modulation time is used for earlier eluents with sharper peak widths—which reduces the chance for undersampling—and a longer modulation time for later eluents to accommodate the generally broader ^2^D peaks that require longer analysis times for separation.

**Fig. 7:**
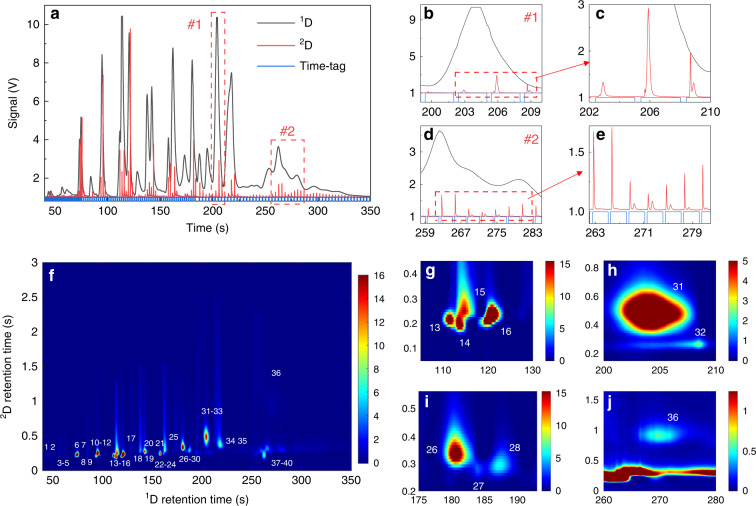
^1^D and ^2^D chromatograms and hybrid 2D contour plot. **a** ^1^D and ^2^D chromatograms of the 40 VOC mix (Table S2) obtained by ^1^D and ^2^D μPIDs in the portable comprehensive 2D μGC device. Magnifications of area #1 (**b**, **c**) and area #2 (**d**, **e**) in ^1^D and ^2^D. **f** Hybrid 2D contour plot using both the ^1^D and ^2^D chromotograms in Fig. (**a**). Magnifications of peaks 13–16 (**g**), 31–32 (**h**), 26–28 (**i**) and 36 (**J**). Panels (**h**) and (**j**) correspond to (**c**) and (**e**), respectively. For comparison, the 2D contour plot using the conventional method that relies only on the ^2^D μPID data is presented in Fig. S 18. Experimental conditions: ^1^D flow rate = 1.2 mL/min; ^2^D flow rate = 11 mL/min. Loading time = 0.4 s; modulation is segmented into three periods: modulation time = 1 s from 0 to 75 s; 2 s from 75 to 180 s; and 4 s from 180 to 350 s. Both ^1^D μcolumn (OV-1) and ^2^D integrated FRPM module undergo temperature ramping (Fig. S3(B) and S17). Helium is used as both ^1^D carrier gas and auxiliary flow

The comprehensive 2D μGC device was employed to separate 40 VOCs (selected such that some have similar boiling points but different polarities to enable ^2^D separation, see Supplementary Table S2) in ~5 min, as shown in Fig. 7. The ^2^D column (i.e., the integrated FRPM module) was operated using a carefully tuned temperature profile (Supplementary Fig. S17) to balance the sharpness of the ^2^D elution peaks while maintaining sufficient ^2^D separation. Figure 7a shows the ^1^D and modulated ^2^D chromatograms obtained by the ^1^D and ^2^D μPIDs, respectively. Two magnified images of certain regions are provided to visualize exemplary additional separations in ^2^D (Fig. 7b–e). For example, in Fig. 7b, c, two VOCs are completely coeluted in ^1^D yet separated in ^2^D. Another coelution example can be seen in Supplementary Fig. S19. Figure 7f presents the hybrid 2D contour plot generated using both the ^1^D and ^2^D chromatograms obtained (see Methods for a brief description of the hybrid contour plot method). Throughout the entire analysis, each analyte is eluted from the ^2^D column within a single modulation cycle, i.e., no wrap-around was observed. The 2D contour plot using the conventional method, which relies only on the ^2^D μPID data, is plotted in Supplementary Fig. S18. By virtue of the additional ^1^D information, more peaks are identified in the same segment (e.g., Fig. 7g, i) compared to the conventional 2D contour plot (e.g., Supplementary Fig. S18b, c). As a result, all 40 VOCs are separated using our hybrid method vs. only 32 peaks using the conventional 2D contour plot method.

Use of the two detectors and the hybrid method significantly benefits ^1^D chromatogram construction due to improved ^1^D peak capacity and accuracy in ^1^D peak retention time. To evaluate the increase in the ^1^D peak capacity, ^1^D retention times and peak widths of benzene, C_7_, C_8_, and C_9_ are extracted from the conventional and hybrid 2D contour plots and are listed in Table 1. All of these analytes from the hybrid 2D contour plot have sharper peak widths than the widths obtained from the conventional 2D contour plot, and their widths (and retention times) are very close to the directly measured values from the ^1^D chromatogram. Notably, the C_9_ peak width is narrower in the hybrid reconstruction than in the measured value due to its coelution in ^1^D (Supplementary Fig. S19a). This suggests that our algorithm can reconstruct the real (i.e., not coeluted) peak for C_9_ by using the ^1^D data. The ^1^D peak capacity of these analytes is calculated using the formula^15^:

1 $$n_p = \mathop {\sum}{\frac{{1.18}}{{R_s}}} \,\,{ \times \left( {\frac{{t_2 - t_1}}{{w_1 + w_2}}} \right)}$$

where $t_1$ and $t_2$ are the retention times for two adjacent peaks and $w_1$ and $w_2$ are the corresponding peak widths (full-widths-at-half-maximum). *R*_*s*_ is the resolution. The ^1^D peak capacity using the hybrid method yields $n_{p\_hybrid} = 28$ (*R*_*s*_ = 1), showing a significant improvement over the conventional method $n_{p\_conv} = 20$. In addition, the accuracy of the ^1^D retention time is improved. For example, the C_7_ peak position is 94.7 s, as measured directly by the ^1^D μPID. The reconstructed peak position is 95 s using our algorithm, compared to 95.6 s using the conventional method.

**Table 1:** ^1^D retention times (RTs) and full-widths-at-half-maximum (FWHMs) for benzene, C_7_, C_8_, and C_9_ reconstructed from conventional (conv, Supplementary Fig. S18a) and hybrid (Fig. 7f) 2D contour plots and measured (meas) directly from the ^1^D chromatogram (Fig. 7a)

	RT_conv_	FWHM_conv_	RT_hybrid_	FWHM_hybrid_	RT_meas_	FWHM_meas_
Benzene	85.7	2.7	84.8	2.6	84	2.4
C_7_	95.6	2.4	95	1.9	94.7	2
C_8_	143.4	2.5	142.6	2.3	142	2.3
C_9_	197.1	7.2	194.2	2.7	194.6	3.8^a^

All values are provided in seconds

^a^Coelution

The ^2^D peak capacity can be estimated as follows assuming isothermal separation^15^:

2 $$n_{p\_2D} = 1 + \frac{{\sqrt N }}{{4R_s}}\ln \left( {\frac{{t_r}}{{t_m}}} \right)$$

where *N* is the theoretical plate number, *t*_*r*_ is the analyte ^2^D retention time, and *t*_*m*_ is the holdup time. Using C_9_ (*t*_*r*_ = 0.261 s, peak width = 0.055 s, reconstructed from the hybrid 2D contour plot) and a holdup time of 0.17 s, $n_{p\_2D} = 2.2$ (*R*_*s*_ = 1). Therefore, the peak capacity of the whole system is 62. Note that in Fig. 7, the comprehensive 2D μGC system is optimized to separate all 40 VOCs in a short time rather than to achieve a high peak capacity.

## Discussion

This article presents the development of a new FRPM for 2D comprehensive GC that allows a high auxiliary flow rate (and hence a high ^2^D flow rate) without disturbing or interrupting the ^1^D flow. The FRPM is shown to enable rapid ^2^D injection and separation while maintaining ^1^D separation and ^1^D peak shape. The integrated flow resistor in the FRPM not only helps reduce the impact of the auxiliary flow on the ^1^D peak shape and elusion time but also reduces the excess consumption of the auxiliary flow.

It should be noted that the ^1^D and ^2^D performance is affected by the auxiliary flow rate. As shown in Fig. 4a, the ^2^D peak width is determined by the auxiliary flow and ^1^D flow rate ratio. A higher auxiliary flow results in a narrower ^2^D injection peak width, which is preferred for ^2^D separations. However, an excessive auxiliary flow rate may cause ^1^D flow fluctuations (i.e., transient pressure changes), which cause ^1^D peak jittering (see Supplementary Figs. S5 and S6) and elution delay (see Supplementary Figs. S4 and S14). Therefore, the auxiliary flow must be kept in a reasonable range such that the ^1^D chromatogram is not disturbed. In our 2D μGC system, the flow rate ratio (when ^1^D flow is fixed at ~1 mL/min) is determined to be optimal at ~10 for narrow injection from ^1^D to ^2^D while avoiding any ^1^D jittering or significant ^1^D elution delay (see Section S2 in the SI). In addition, μPID responds to analyte concentration, not mass flow rate. Therefore, the auxiliary flow rate (or ^2^D carrier gas flow rate) or any pressure changes caused by valve switching do not affect the PID sensitivity. No fluctuations or spikes in the PID signal are observed when the valves are actuated.

In the FRPM, the duty cycle (i.e., the loading time vs. modulation time) ranges from 10 to 50% as a diverting flow modulation (e.g., 0.2–1.0 s loading time in a 2 s modulation cycle), which is low compared to that of other valve-based differential flow modulators, where a duty cycle as high as 80% was used^19–21^ (note that stop-flow modulation can achieve a 100% duty cycle). A longer loading time would allow increased mass transfer to ^2^D but would also lead to a broader ^2^D injection peak width and a shorter allowed ^2^D separation time. Although this low duty cycle does not affect the present 2D μGC system due to the use of concentration-dependent vapor sensors (i.e., μPIDs), the ^2^D signal (i.e., ^2^D detector’s sensitivity) may be reduced if the ^2^D detector (e.g., flame ionization detector) depends on the mass flow rate.

We subsequently used the integrated FRPM to construct a first-of-its-kind automated portable comprehensive 2D μGC. Rapid separation of a diverse set of 40 VOCs in ~5 min is demonstrated. Due to the FRPM, an undisturbed ^1^D chromatogram can be obtained and allows for the development of a new algorithm to construct hybrid 2D contour plots incorporating both ^1^D and ^2^D chromatograms. This results in more accurate ^1^D peak reconstructions and increased peak capacities compared to those of the conventional method, which uses only the ^2^D data. 32 peaks were counted from the conventional 2D contour plot (Supplementary Fig. S18a) and 29 peaks from the ^1^D chromatogram alone (Fig. 7a) compared to the 40 peaks separated by the hybrid 2D contour plot (Fig. 7f), corresponding to a gain of 8 and 11 peaks compared to those of a comprehensive 2D GC using the conventional method and a single column 1D GC, respectively.

If desired, the FRPM (and hence the comprehensive 2D μGC) can be operated in stop-flow mode^15^ by permanently closing the waste line valve and letting the ^1^D and auxiliary flow share the same pressure/flow source. Supplementary Fig. S20 shows the separation of the same 40 VOCs in Fig. 7 using this mode. The ^1^D separation time and peak width are both significantly increased with strong ^1^D flow perturbations. While this is a drawback compared to the current operating paradigm, the FRPM’s flexibility of operation allowing for stop-flow mode may be useful for some applications, such as those where rapid analysis is not crucial.

Compared to other pneumatic modulators, the major advantages of the FRPM are as follows: (1) Sharp ^2^D injection and rapid ^2^D separation with a high ^2^D flow rate (or high ^2^D/^1^D flow rate ratio) are enabled. (2) Continuous and undisturbed ^1^D separation concomitant with ^2^D separation is enabled, which facilitates a new method for constructing hybrid 2D contour plots using both ^1^D and ^2^D chromatograms, thus enhancing the overall 2D GC peak capacity without sacrificing the analysis time. (3) Integration with a ^2^D column is possible, reducing the footprint to be amendable for μGC. In contrast, most flow modulators are bulky and only suitable for benchtop operation^19–23^. However, due to its nature as a diverting flow modulator, the FRPM has an inherent disadvantage of mass loss due to the low duty cycle (20–50%) compared to that of most differential flow modulators, which can achieve a high duty cycle^20,21^. A detailed comparison of different types of pneumatic modulators is given in Supplementary Table S3.

The FRPM described here is applicable to traditional comprehensive 2D GC in which the two columns are connected in series (via a modulator) and whose total peak capacity is multiplicative. It can also be used in a heart-cutting 2D GC. There is another type of 2D GC (pseudo-2D GC) device^24^, in which multiple columns are arranged in parallel and coated with different stationary phases of varying polarities so that analytes of the same boiling point but different polarities can be separated. The FRPM is not applicable to this type of GC.

In summary, we developed a first-of-its-kind automated portable comprehensive 2D μGC using an integrated FRPM. This compact and versatile device provided portable stand-alone separations of 40 VOCs in ~5 min with an enhanced peak capacity compared to that of the conventional 2D GC. Further integration of the FRPM with both ^1^D and ^2^D μcolumns can further improve device compactness, potentially facilitating a hand-held device applicable to many more field applications.

## Materials and methods

### Materials

Analytical standard-grade hexane, heptane, octane, benzene, toluene, hexamethyldisilazane (HMDS), and the 40 VOCs listed in Supplementary Table S2 were purchased from Sigma-Aldrich (St. Louis, MO). N-type silicon wafers (P/N 1095, 100 mm diameter, 500 μm thickness), P-type heavily doped wafers (100 mm diameter, 0.001–0.005 Ω-cm, 400 μm thickness), and Borofloat 33 glass (P/N 517) were purchased from University Wafer. Carbopacks B (P/N 20273) and X (P/N 10437-U) were purchased from Sigma-Aldrich. Additional accessory materials are provided in Supplementary Table S4. All materials were used as purchased without further purification or modification. Helium (99.5% purity, P/N 49615He) was used as the carrier and auxiliary gas and was purchased from Leland Gas Technologies (South Plainfield, NJ).

### Component fabrication

The 10 m ^1^D μcolumn (cross-section: 200 μm × 250 μm, width × depth), the stand-alone FRPM, and the integrated FRPM and 0.5 m ^2^D μcolumn were fabricated according to the fabrication process depicted in Supplementary Fig. S1. The stand-alone FRPM had no heater on the backside of the chip, but the integrated FRPM and ^2^D μcolumn were fabricated with a shared backside heater. The fabrication yield for the stand-alone FRPM was >95% (132 chips per 4-inch wafer), >90% for the integrated FRPM (12 chips per 4-inch wafer), and >50% for the 10 m μcolumn (2 chips per 4-inch wafer).

The integrated FRPM with a 0.5 m ^2^D μcolumn (cross-section: 250 μm × 250 μm) coating procedure is depicted in Supplementary Fig. S2. Prior to coating, both the ^2^D μcolumn and FRPM channels were deactivated by eight repeated injections of HMDS at 120 °C over 1 h. The coating outlet was blocked with a rubber septum during deactivation. During ^2^D μcolumn coating, the outlets of the FRPM were blocked, leaving only the coating outlet open to ensure that no coating solution flowed into the FRPM channels. A dummy 10 m μcolumn was attached to the coating outlet as a flow resistor to control the coating flow speed. The ^2^D μcolumn was dynamically coated with PEG by injecting 15 μL of solution and pushing it out at a rate of 5 cm/min. PEG: 2% (w/w) solution of CarboWAX in dichloromethane with azobisisobutyronitrile (1% w.r.t. CarboWAX) as a crosslinker. The coating process was repeated 2 times. The column was subsequently treated with HMDS after each coating and then baked at 180 °C for 1 h prior to use. Finally, the guard column attached to the coating outlet was removed, and hysol epoxy was applied to block the outlet. The 10 m μcolumn underwent the same coating procedure with a 3% (w/w) solution of OV-1 in dichloromethane. The resistance of the integrated heater was measured to be 40 Ω for the integrated FRPM chip and 28 Ω for the 10 m μcolumn. Both columns were wire bonded to PCB boards to allow for pulse-width-modulated heating using a peak voltage of 24 V. The μPID chip was fabricated as described in our previous work^25,26^. The μPID array was packaged on a PCB board, as shown in Fig. 6b.

The stainless steel preconcentrator was made by first cutting a 21.5-gauge stainless steel tube to 3.5 cm in length. One end was first plugged with glass wool. Subsequently, the tube was filled with 0.75 mg of Carbopack B, followed by 0.75 mg of Carbopack X, and the other end was then plugged with glass wool again. Two universal press-tight connectors were attached to both ends of the stainless steel tube after loading and fixed using hysol epoxy. A very thin layer of epoxy (∼0.2 mm) was also applied to the outer surface of the stainless steel tube body. The entire preconcentrator was placed into an oven at 120 °C and left to dry for 12 h. Finally, Kapton tape was wrapped around the stainless steel tube before it was wrapped with a 32-gauge nickel chromium heating wire (resistance ∼7 Ω) to ensure electrical isolation between the stainless steel tube and heating wires.

### Computational fluid dynamics simulation

COMSOL Multiphysics^®^ was used to generate the results presented in Fig. 2. A laminar flow module was used in the simulation, where helium was used as the gas flow and silicon was used as the walls. Closed valves were simulated by assigning an extremely large viscosity (i.e., 10,000) at a short portion of the inlet (Port 1) and the waste line (Port 4) simultaneously.

### Comprehensive 2D μGC system setup and operation

The comprehensive 2D μGC system consisted of a stainless steel preconcentrator, a 10 m OV-1 coated ^1^D μcolumn, an integrated FRPM and a 0.5 m ^2^D WAX μcolumn, and two flow-through μPIDs at the end of ^1^D and ^2^D, respectively. Components were interconnected using universal press-tight connectors and deactivated fused silica capillaries. A detailed schematic along with a device photograph is shown in Fig. 6b. The ^1^D flow rate was calibrated at the end of the ^2^D μPID (Port 3) with both valves closed. The ^2^D flow rate was calibrated at the end of the ^2^D μPID by opening both valves at the auxiliary flow inlet (Port 1) and waste line (Port 4). Analytes were stored in a Tedlar bag and sampled into the preconcentrator before backflush injection into the ^1^D μcolumn. During operation, the analytes were separated by the ^1^D column, flowed through the ^1^D μPID, and subsequently entered the FRPM module for 2D comprehensive modulation and separation. Separation was conducted using temperature ramped programming in both dimensions via the integrated backside heaters. Helium (99.5% purity) was used as the carrier and auxiliary gas. Loading and modulation times were set by simultaneously controlling the valves’ ON and OFF states at the auxiliary flow inlet (Port 3) and waste line (Port 4).

Segmented modulations were achieved by assigning different loading and modulation times to different segments of analysis. The current work used modulation times of 1 s from 0 to 75 s, 2 s from 75 to 180 s, and 3 s from 180 to 350 s. The loading time was kept at 0.4 s during all segments. Portable μGC operation was controlled by LabVIEW^TM^ software developed in-house.

### 2D chromatogram construction

The 2D contour plots in Supplementary Fig. S18 were constructed with the traditional method adopted in conventional comprehensive 2D GC that has only one detector at the outlet of the ^2^D column (i.e., no detector at the end of the ^1^D column). They were generated through the 2D interpolation of the original 2D GC data based on a cubic spline^27^. The interpolated value at a query grid point was based on a cubic interpolation of the values at neighboring grid points in each respective dimension.

The hybrid 2D contour plot in Fig. 7f–j was constructed with the signal obtained from both ^1^D and ^2^D μPIDs. The traditional interpolation method based on a cubic spline was first applied using the ^2^D GC data. ^1^D GC data were then adopted to correct the contour data along the ^1^D direction, while peak shapes along the ^2^D direction were preserved. The details of the algorithm will be presented in a separate paper.

## Supplementary Information

Supplementary Information
