# Supplementary material for: Portable comprehensive two-dimensional micro-gas chromatography using an integrated flow-restricted pneumatic modulator
Source: Microsyst Nanoeng. 2022 Nov 1;8:115. doi: 10.1038/s41378-022-00452-5 (PMC9622416; doi:10.1038/s41378-022-00452-5)
Supplement: Supplementary file 1 — Supplementary Information [file 41378_2022_452_MOESM1_ESM.docx]

**Supplementary Information to**

**Portable Comprehensive Two-Dimensional Micro-Gas Chromatography Using an Integrated Flow-Restricted Pneumatic Modulator**

Xiaheng Huang^1,2,3,4^, Maxwell Wei-hao Li^1,2,3,4^, Wenzhe Zang^1,3^, Xiaolu Huang^1,3,4^,

Anjali Devi Sivakumar^1,2,3,4^, Ruchi Sharma^1,3,4^, and Xudong Fan^1,3,4*^

^1^Department of Biomedical Engineering,

University of Michigan, Ann Arbor, MI 48109, USA

^2^Department of Electrical Engineering and Computer Science,

University of Michigan, Ann Arbor, MI 48109, USA

^3^Center for Wireless Integrated MicroSensing and Systems (WIMS^2^),

University of Michigan, Ann Arbor, MI 48109, USA

^4^Max Harry Weil Institute for Critical Care Research and Innovation

University of Michigan, Ann Arbor, MI 48109, USA

*: Corresponding author

**S1. Fabrication and coating of modulators and columns**

**Figure S1.** FRPM (including integrated version that has a ^2^D column) and μcolumn microfabrication process. (a) Soft mask of photoresist exposing both column and inlets/outlets. (b) Creation of an oxide hard mask through DRIE (deep-reactive-ion-etching). (c) Soft mask exposing only inlets/outlets for DRIE to 150 μm. (d) DRIE on the entire pattern area to etch inlets/outlets to 400 μm and column to 250 μm. (e) BHF (buffered hydrofluoric acid) strip off oxide mask and anodic bonding with Pyrex glass to seal the column. (f) Patterned metal heater (30 nm Titanium/320 nm Platinum) deposition on the backside.

**Figure S2.** Coating procedure for the integrated FRPM with a ^2^D μcolumn (A) Step I: Hexamethyldisilane (HMDS) deactivation of all microfluidic channels. (B) Step II: ^2^D μcolumn coating. A dummy 10 m μcolumn was used for coating flow control. Only channels labelled red were coated. After coating, the coating outlet was sealed by epoxy.

**S2. Characterization of FRPM module**

Figure S3 shows the unmodulated operation for the system depicted in Figure 3(A). The height equivalents to theoretical plates (HETPs) for the 10 m OV-1 ^1^D μcolumn is listed in Table S1.

**Figure S3.** (A) Unmodulated operation for calibration of ^1^D and ^2^D μPIDs using the setup in Figure 3(A). ^1^D = ^2^D = 1.2 mL/min. It shows that the ^2^D μPID was 2.4 times more sensitive than ^2^D μPID. (B) Temperature ramping profile of the ^1^D 10 m OV-1 coated μcolumn.

Figures S4-S11 provide additional characterization of FRPM in terms of the maximally allowed ^2^D/^1^D flow ratio and peak height (and peak area) for different loading times, as well as the comparison between the modulator with and without the flow resistor.

As mentioned above, a high flow rate ratio is desired in order to generate a sharp ^2^D injection peak and expedite ^2^D separation. However, at an excessive flow rate ratio (*i.e*., strong auxiliary flow), the ^1^D flow is slowed down or even pushed backwards, causing delay in ^1^D retention time measured by ^1^D μPID (see Figure S4) and jittering in ^1^D chromatogram (see Figure S5). Delay in retention time prolongs the analysis time and reduces the peak capacity and jittering makes ^1^D chromatogram analysis (such as peak fitting and apex identification) nearly impossible. The flow resistor (*i.e*., the narrow channel) in the FRPM significantly mitigates the ^1^D retention time delay and jittering at a high flow rate ratio.

According to Figure S4, no significant delay in the ^1^D eluent’s retention time was observed compared to the unmodulated case. For example, ^1^D retention time for C_7_ was 120 s and 100 s, respectively, for modulated (at a flow rate ratio of 20) and unmodulated operation (see Figure S3(A)). Additionally, according to Figure S5, even at the flow rate ratio of 17 (^2^D flow = 20 mL/min), the ^1^D chromatogram is still well-behaved and smooth. The jittering does not emerge until when the flow rate ratio is above 20 (^2^D flow rate = 25 mL/min). In contrast, when the modulator was operated without the 40 μm wide flow resistor (*i.e*., the flow resistor’s channel width became 250 μm rather than 40 μm – see Figure S6(A)), not only the ^1^D retention time was significantly delayed (for example, C_7_ retention time became 145 s at the same flow rate ratio of 20), but the ^1^D peak was strongly perturbed (see Figure S6(C)-(D)) at a low flow rate ratio (such as 9 when ^2^D flow = 11 mL/min).

**Figure S4.** ^1^D peak delays of C_6_, C_7_, and C_8_ detected by ^1^D μPID vs. flow rate ratio (A), loading time (B), and modulation time (C). Experimental conditions: (A) and (C) loading time = 0.25 s. (B) loading time = 0.1 to 0.5 s. (A) and (B) modulation time = 2 s. (C) modulation time = 1 to 4 s. (A) ^2^D flow rate = 4 to 40 mL/min. (B) and (C) ^2^D flow rate = 16 mL/min. For all experiments, ^1^D flow rate = 1.2 mL/min. Error bars are obtained with 3 measurements. The results agree qualitatively with the theoretical calculations shown in Figure S7.

**Figure S5.** Zoom-in ^1^D and ^2^D chromatograms of C_7_ using the FRPM module. ^2^D flow rate = 14 mL/min (A), 25 mL/min (B), 30.5 mL/min (C), and 37 mL/min (D). For all experiments, ^1^D flow rate = 1.2 mL/min, loading time = 0.25 s, and modulation time = 2 s. Black arrows indicate the jittering in a ^1^D peak.

**Figure S6.** (A) Schematic of a microfabricated pneumatic modulator without the 40 μm wide flow resistor. The flow resistor region has the same cross section of 250 μm x 250 μm (width x depth) as all other channels. (B)-(D) Zoom-in ^1^D and ^2^D chromatograms of C_7_. ^2^D flow rate = 6 mL/min (B), 11 mL/min (C), and 20 mL/min (D). For all experiments, ^1^D flow rate = 1.2 mL/min, loading time = 0.25 s, and modulation time = 2 s. Black arrows indicate the jittering in a ^1^D peak.

To better understand the delay in ^1^D retention time, let us first assume that the analyte speed in the ^1^D column during unmodulated operation (*i.e*., both valves in Figure 1(A) are closed) is V_0_, which is also the analyte speed in the ^1^D column when the analyte is transferred from ^1^D to ^2^D during the loading state under modulated operation when both valves are closed. During the ^2^D separation stage, a high auxiliary flow is served when both valves are open. The analyte speed in the ^1^D column is reduced to αV_0_, where α ranges from 0 to 1. Let us further assume that the modulation time is t and the duty cycle (the ratio of the loading time and the modulation time) is m, then the analyte effective speed in the ^1^D column becomes

$$V_{eff}=V_{0}\times m+\alpha V_{0}\times\left( 1-m \right) (1)$$

The analyte retention time is

$$T=\frac{L}{V_{eff}}=\frac{L}{V_{0}\times m+\alpha V_{0}\times(1-m)} (2)$$

Now we can study a few scenarios.

1. Unmodulated operation. In this case, m = 1, the analyte ^1^D retention time is T = L/V_0_.

2. Stop-flow operation. In this case, α = 0, m ranges from 0-1. Consequently, the 1D retention time becomes T = L/(mV_0_) and is significantly delayed as compared to unmodulated operation. For example, when m = 0.25, the ^1^D retention times becomes 4 times longer.

3. Modulated operation with our pneumatic modulator. In Figure S7, we plot the ^1^D retention time delay with different α values (*i.e*., different flow rate ratios), loading times, and modulation times. In all calculations below, we fix L = 10 m and V_0_ = 0.1 m/s.

**Figure S7.** (A) Retention time delay calculated from Eq. (2) as a function of α. Note that a higher α value corresponds to a smaller flow rate ratio. Loading time = 0.25 s, modulation time = 2 s, m = 0.125. (B) Retention time delay as a function of loading time. α = 0.8, modulation time = 2. (C) Retention time delay as a function of modulation time. α = 0.8, loading time = 0.25 s. In all calculations, L = 10 m and V_0_ = 0.1 m/s.

In Figures S8 and S9, we also examine the peak area (and height) for different loading times, as the loading time determines the amount of mass transferred from ^1^D to ^2^D. It is shown in Figure S7 that only small peaks emerge in ^2^D when the loading time is 0.1 s. However, the peak height increases significantly when the loading time is above 0.2 s. Since the peak height varies depending on the time when the loading from ^1^D to ^2^D occurs, we decide to use the entire ^2^D peak area corresponding to the same analyte peak in ^1^D (there are multiple ^2^D peaks for a given ^1^D peak) to estimate the total mass transfer. As expected, Figure S9(A) shows that the ^2^D peak area, which is normalized by the corresponding ^1^D peak area, increases linearly with the loading time. In Figure S9(B), we further normalized the ^2^D peak area by the loading time. It is found the loading time has a threshold of ~0.2 s, above which the mass transfer is nearly the same regardless of the loading time. However, below 0.2 s, the mass transfer is reduced significantly. This threshold behavior may be attributed to the minimal time required to re-establish the pressure to push the ^1^D eluent through the narrow channel flow resistor when the two 2-port valves are switched from open to close. Similar threshold behavior is observed with the pneumatic modulator without the 40 μm wide flow resistor, *i.e*., the channel width is 250 μm (see Figures S10 and S11). The threshold is reduced to approximately 0.05 s, since it is easier (and quicker) to re-establish the pressure to push the ^1^D eluent through a wider (250 μm) channel.

Note that we also microfabricated and tested pneumatic modulator chips with the flow resistor’s width varying from 20 μm to 250 μm. The 40 μm wide flow resistor provides the optimal performance in terms of the maximally allowed ^2^D/^1^D flow rate ratio (without causing ^1^D peak distortion and significant ^1^D retention time delay) and ^2^D injection width.

**Figure S8.** Zoom-in ^1^D and ^2^D chromatograms of C_7_ using the FRPM module. Loading time = 0.1 s (A), 0.2 s (B), 0.3 s (C), 0.4 s (D), and 0.5 s (E). For all experiments, modulation time = 1 s, ^1^D flow rate = 1.2 mL/min, and ^2^D flow rate = 16 mL/min.

 **Figure S9.** (A) Peak area ratio of between C_7_ peaks in ^2^D and ^1^D extracted from Figure S8. (B) Peak area ratio normalized by the loading time extracted from (A). Error bars are obtained with 3 measurements.

**Figure S10.** Zoom-in ^1^D and ^2^D chromatograms of C_7_ operated without a flow resistor (*i.e*., the channel width is 250 μm) with a loading time = 0.025 s (A), 0.05 s (B), 0.1 s (C), 0.2 s (D), 0.3 s (E), 0.4 s (F), and 0.5 s (G). For all experiments, modulation time = 1 s, ^1^D flow rate = 1.3 mL/min, and ^2^D flow rate = 7.5 mL/min. Black arrows indicate the jittering features in ^1^D peak.

**Figure S11.** (A) Peak area ratio of between C_7_ peaks in ^2^D and ^1^D extracted from Figure S10. (B) Peak area ratio normalized by the loading time extracted from (A). Error bars are obtained with 3 measurements.

**S3. Single-valve FRPM module**

**Figure S12.** Single-valve based FRPM operating principle for comprehensive 2D μGC. (A) ^1^D to ^2^D loading configuration with the normally-open (NO) port open in the 3-port valve (typical ^1^D flow rate: ~1 mL/min). C = common; NC = normally-closed. The flow resistor (labelled orange) is a 1.2 m long guard column. The blue and green arrows depict the ^1^D and auxiliary flow directions, respectively. (B) ^2^D separation with the NC port open for a high ^2^D flow (typical flow rate: ~10 mL/min), enabling sharp ^2^D injection and rapid ^2^D separation. (C) Single-valve FRPM schematic. It has an additional Port 5 compared to FRPM in Figure 1. (D) Photograph of the single-valve FRPM module with a 3-port valve.

**Figure S13.** (A) Setup used to characterize the single-valve FRPM module (identical to the one in Figure 3(A)). (B) ^1^D and ^2^D chromatograms of C_6_, C_7_, C_8_, benzene, and toluene. (C) Zoom-in of C_7_ in ^1^D and ^2^D. (D) Zoom-in of C_7_ in ^2^D. Experimental conditions: loading time = 0.25 s, modulation time = 2 s, ^1^D flow rate = 1.4 mL/min, and ^2^D flow rate = 18.5 mL/min. The ^1^D flow rate was calibrated at the end of the ^1^D μPID before connecting to the single-valve FRPM module. The ^2^D flow rate was calibrated at the end of the ^2^D μPID after connecting the single-valve FRPM module and switching the 3-port valve to the normally closed port. The ^1^D μcolumn underwent temperature ramping (see Figure S3(B)). The single-valve FRPM was at room temperature (~20 ^o^C). Helium was used as both ^1^D carrier gas and auxiliary flow. Note that an external flow resistor (1.2 m guard column) was used to restrict the buffer flow rate during loading but can in principle be microfabricated on the same chip for compactness.

**Figure S14.** (A) ^2^D peak widths; (B) deviations of ^2^D peak widths from ideal injection widths; and (C) ^1^D peak delay of C_6_, C_7_, and C_8_ vs. flow rate ratio of the single-valve FRPM module-based system. Experimental conditions: ^2^D flow rate = 3 to 44 mL/min, ^1^D flow rate = 1.4 mL/min, loading time = 0.25 s, and modulation time = 2 s for all experiments. Error bars are obtained with 3 measurements.

**S4. Characterization of integrated FRPM and ^2^D column**

**Figure S15.** (A) ^2^D peak widths extracted from Figure 5(C). (B) Deviations of ^2^D peak widths from ideal ^2^D injection widths. (C) ^1^D peak delay of C_6_, C_7_, and C_8_ vs. flow rate ratio of the integrated FRPM module based system. Experimental conditions: ^1^D flow rate = 1.1 mL/min, ^2^D flow rate = 3.6 to 31 mL/min, loading time = 0.25 s, and modulation time = 2 s for all experiments. Error bars are obtained with 3 measurements.

**Figure S16.** Zoom-in ^1^D and ^2^D chromatograms of C_7_ using the FRPM module based portable comprehensive 2D μGC with ^2^D flow rate = 9 mL/min (A), 14 mL/min (B), 20 mL/min (C), and 25 mL/min (D). For all experiments ^1^D flow rate = 1.1 mL/min, loading time = 0.25 s, and modulation time = 2 s. Black arrows indicate the jittering features in a ^1^D peak when the ^2^D flow rate is above 20 mL/min.

**S5. Integrated FRPM based comprehensive 2D μGC device**


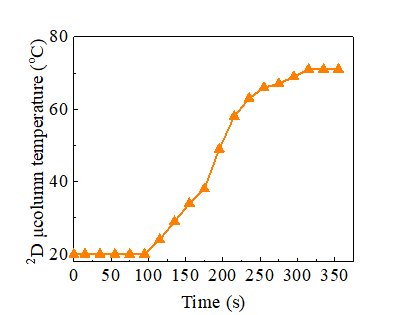


**Figure S17.** Temperature ramping profile of the integrated FRPM chip. The ^2^D column has the same temperature ramping profile.

**Figure S18.** (A) 2D contour plot generated using only the ^2^D chromatogram in Figure 7(A). (B) and (C) correspond to the zoom-in areas of Figures 7(G) and (I), respectively. Note that only 32 peaks were counted from the conventional 2D contour plot in (A) vs. 40 peaks from the hybrid 2D contour plot in Figure 7(F).

**Figure S19.** (A) Zoom-in of C_9_ from Figure 7(A). Inlet shows the ^2^D separation of 2 VOCs, implying co-elution in ^1^D. (B) The corresponding 2D contour plot from Figure 7(F).

**Figure S20.** (A) Schematic of the FRPM based comprehensive 2D μGC device operated in a stop-flow modulation mode, in which Port 4 is permanently blocked. When the 2-port valve is closed, ^1^D separation takes place and the eluents from the ^1^D column are loaded to the ^2^D column. When the 2-port valve is open, ^1^D separation is suspended and the helium source generates a high ^2^D flow for rapid ^2^D separation. (B) ^1^D and ^2^D chromatograms of 40 VOCs generated by stop-flow modulation. Inlet shows the ^2^D separation of 2 VOCs. The entire separation is completed in ~600 s, much longer than 300 s reported in Figure 7(A). (C) Zoom-in portion of (B), where strong jittering in the ^1^D chromatogram can be seen easily. (D) Unmodulated operation when the 2-port valve is permanently closed. Experimental conditions: ^1^D flow rate = 1.2 mL/min, ^2^D flow rate = 11 mL/min, loading time = 0.4 s, and modulation time = 2 s. Unmodulated: ^1^D flow rate = ^2^D flow rate = 1.2 mL/min. Both ^1^D μcolumn and ^2^D integrated FRPM module underwent temperature ramping shown in Figures S3(B) and S17, respectively.

Table S1. Height equivalents to theoretical plates (HETPs) for 10 m OV-1 ^1^D μcolumn at a flow rate of 40 cm/s. Data are calculated from the ^1^D chromatogram in Figure S3(A).

| Analyte | RT (s) | FWHM (s) | N | HETP (mm) |
| --- | --- | --- | --- | --- |
| C_6_ | 61.1 | 1.46 | 9711.32 | 1.03 |
| Benzene | 82 | 1.97 | 9607.20 | 1.04 |
| C_7_ | 97.1 | 1.6 | 20422.08 | 0.49 |
| Toluene | 128.2 | 2.9 | 10836.31 | 0.92 |
| C_8_ | 143.3 | 2.5 | 18218.55 | 0.55 |

Table S2. 40 VOCs used in the comprehensive 2D μGC system.

| # | Analyte | Boiling point (^o^C) | Dipole moment (D) |
| --- | --- | --- | --- |
| 1 | acetone | 56.2 | 2.85 |
| 2 | 2-methylfuran | 64 | 0.72 |
| 3 | tetrahydrofuran (THF) | 66 | 1.63 |
| 4 | trans-2-hexene-1-al | 68 | N.A. |
| 5 | hexane (C_6_) | 69 | 0 |
| 6 | ethyl acetate | 77 | 1.78 |
| 7 | 1-chlorobutane | 78 | 1.9 |
| 8 | benzene | 80.1 | 0 |
| 9 | cyclohexane | 80.75 | 0 |
| 10 | isopropanol | 82.5 | 1.66 |
| 11 | heptane (C_7_) | 98 | 0 |
| 12 | 2-pentanone | 101 | N.A. |
| 13 | 1,4-dioxane | 101 | 0.45 |
| 15 | methylcyclohexane | 101 | 0 |
| 14 | 3-pentanone | 102 | N.A. |
| 16 | toluene | 110.6 | 0.36 |
| 17 | methyl isobutyl ketone | 117 | 2.8 |
| 18 | pinacolyl alcohol | 120.4 | N.A. |
| 19 | octane (C_8_) | 125.6 | 0 |
| 20 | butyl acetate | 126.1 | 1.87 |
| 21 | 2-hexanone | 128 | 2.69 |
| 22 | hexanal | 129 | N.A. |
| 23 | chlorobenzene | 132 | 1.5 |
| 24 | 1-chlorohexane | 135 | 1.94 |
| 25 | ethylbenzene | 136 | 0.58 |
| 26 | 1-pentanol | 138 | 1.7 |
| 27 | xylene | 138.3 | 0 |
| 28 | styrene | 145 | 0.181 |
| 29 | 2-heptanone | 150 | 2.59 |
| 30 | nonane (C_9_) | 151 | 0.07 |
| 31 | cumene | 152 | 0.65 |
| 32 | heptanal | 155 | 2.56 |
| 33 | 1-hexanol | 157 | 1.6 |
| 34 | decane (C_10_) | 174.1 | 0.07 |
| 35 | dipentene | 176 | N.A. |
| 36 | benzaldehyde | 178.1 | 2.89 |
| 37 | benzyl chloride | 179 | 1.74 |
| 38 | 1,2-dichlorobenzene | 180.5 | 2.5 |
| 39 | 2-nonanone | 195.3 | N.A. |
| 40 | undecane (C_11_) | 196 | 0 |

N.A.: data is not available.

|  | **Modulation mechanism:**  **Diverting and differential** | **Miniaturization**  **and integration on a chip** | **Analyte concentration dilution after transfer** | **Duty cycle and mass loss** | **High flow rate ratio (^2^D/^1^D) without disturbing ^1^D flow** | **Sharp injection and rapid separation without disturbing ^1^D flow** |
| --- | --- | --- | --- | --- | --- | --- |
| **4- or 6-port valve based^1-4^** | Can be either | No | No | Up to 100%. Mass loss varies depending on the duty cycle | Yes | Yes |
| **Regular Deans switch^5^** | Diverting | Possible | Yes | Varies. Mass loss | No (see Note 1) | No |
| **Stop-flow^6^** | Differential | Possible | No | Varies.  No mass loss | No (see Note 2) | Yes |
| **Agilent capillary flow technology (CFT)^7^** | Differential | No | No | No mass loss | No | No |
| **Shimadzu Deans switch^8^** | Diverting | No | Yes | Used in heart-cutting  Mass loss | Possible | Possible |
| **FRPM (this work)** | Diverting | Yes | No | 10-50%. Mass loss  (see Note 3) | Yes | Yes |

Table S3. Comparison of different types of pneumatic modulators.

Note 1: A low ^2^D/^1^D can be used. But at a high ^2^D/^1^D flow rate ratio strong ^1^D disturbances are observed. See our results in Figure S6.

Note 2: Switching between stop and flow causes strong ^1^D flow disturbances. See our results in Figure S20.

Note 3: FRPM can also be used as a stop-flow modulator (see the results in Figure S20).

The use of flow resistors is a common practice in a pneumatic modulator. As pointed out by Seeley^9^, most of such flow resistors are implemented to achieve a desired flow balance during valve-switching for directing the auxiliary flow path for ^1^D to ^2^D injection and ^2^D separation respectively. In the Shimadzu’s flow modulator^8,9^, one of the flow resistors is added between ^1^D and ^2^D to minimize the flow disturbances in the ^1^D column, which serves the same purpose as the flow resistor used in the FRPM (see the detailed comparison in Table S3).

Table S4. Accessory materials for the system assembly.

| Item | P/N | Company |
| --- | --- | --- |
| Norland optical adhesive 68T | 68T01 | Norland |
| Hysol 1C Epoxy | 1373425 | Ellsworth Adhesive |
| Deactivated fused silica tubing | 10010 | Restek |
| Universal press-tight connectors | 20401 | Restek |
| 21.5-gauge stainless steel tubing | 8988K54 | McMaster-Carr |
| Glass wool | 20411 | Sigma Aldrich |
| 32-gauge nickel chromium wire | 32BNC | Consolidated |
| Krypton lamp for PID | 043-257 | MOCON baseline |
| PCB board | Not applicable | M.A.K.S. |
| 3-port valve | LFRA1220170D | Lee Company |
| 3-port valve | LHDA1231315H | Lee Company |
| 2-port valve | LHDB1252115H | Lee Company |
| Pump | T3HP-1PD-12-1SNP | Parker Hannifin |
| Transformer for PID excitation | CCFL FL3209 | Coil Craft |
| DC to DC convertor | 78B12 | Digikey |
| Copper mesh | B08PT35XRD | Amazon |
| Power supply | 1866-RPS-120S-24-ND | Digikey |
| Tedlar bag | 22952 | Restek |

1 Tranchida, P. Q., Purcaro, G., Dugo, P. & Mondello, L. Modulators for comprehensive two-dimensional gas chromatography. *Trends Anal. Chem.* **30**, 1437-1461 (2011).

2 Edwards, M., Mostafa, A. & Górecki, T. Modulation in comprehensive two-dimensional gas chromatography: 20 years of innovation. *Anal. Bioanal. Chem.* **401**, 2335-2349 (2011).

3 Seeley, J. V., Kramp, F. & Hicks, C. J. Comprehensive Two-Dimensional Gas Chromatography via Differential Flow Modulation. *Anal. Chem.* **72**, 4346-4352 (2000).

4 Wang, F. C.-Y. New valve switching modulator for comprehensive two-dimensional gas chromatography. *J. Chromatogr. A* **1188**, 274-280 (2008).

5 Seeley, J. V., Micyus, N. J., Bandurski, S. V., Seeley, S. K. & McCurry, J. D. Microfluidic Deans Switch for Comprehensive Two-Dimensional Gas Chromatography. *Anal. Chem.* **79**, 1840-1847 (2007).

6 Whiting, J. J. *et al.* A high-speed, high-performance, microfabricated comprehensive two-dimensional gas chromatograph. *Lab Chip* **19**, 1633-1643 (2019).

7 Quimby, B., McCurry, J. & Norman, W. Capillary flow technology for gas chromatography: reinvigorating a mature analytical discipline. *LCGC The Peak* **25**, 7-15 (2007).

8 Sciarrone, D. *et al.* Evaluation of tea tree oil quality and ascaridole: a deep study by means of chiral and multi heart-cuts multidimensional gas chromatography system coupled to mass spectrometry detection. *J. Chromatogr.. A* **1217**, 6422-6427 (2010).

9 Seeley, J. V. Recent advances in flow-controlled multidimensional gas chromatography. *J. Chromatogr. A* **1255**, 24-37 (2012).

**References:**
